# Advanced immunology in aging population: unveiling the complexities of vaccine responsiveness

**DOI:** 10.3389/fragi.2025.1682763

**Published:** 2025-11-12

**Authors:** Yan Han, Yanhao Huang, Yuanyu Zhou, Wanke He, Gaomei Luo, Liu Yang, Yanbin Chen, Yiqi Zhu, Wei Jiang, Chanchan Xiao, Jianhui Yan

**Affiliations:** 1 Affiliated Hospital of Xiangnan University, Chenzhou, Hunan, China; 2 The First Affiliated Hospital, Jinan University, Guangzhou, Guangdong, China; 3 Clinical College of Xiangnan University, Chenzhou, Hunan, China

**Keywords:** aging population, immunology, vaccine responsiveness, inflammaging, adjuvant

## Abstract

With the accelerating global population aging, vaccine responsiveness in older adults has emerged as an increasingly critical issue. This review systematically explores age-related changes in immune system function and their impacts on vaccine efficacy. Firstly, we outline the characteristics of immunosenescence and its regulatory effects on vaccine effectiveness from three perspectives: cellular, molecular, and signaling pathway levels. Secondly, we summarize methods for predicting vaccine immune responsiveness (such as biomarkers and advanced immunological assays) and current mainstream strategies for enhancing vaccine immune responsiveness, while enumerating several prominent novel vaccine formulations targeting the older adult population. Finally, we discuss existing controversies and future research directions regarding the study of vaccine responsiveness in older adults, and comprehensively evaluate the current research status of vaccine responsiveness in this demographic. By synthesizing extensive evidence, this review aims to provide new insights into addressing the challenges of vaccinating the older adult population and lay a theoretical foundation for developing more effective immunization strategies tailored to this vulnerable group.

## 1 Introduction

The older adults are at a significantly increased risk of vaccine - preventable diseases (VPDs), which contribute to substantial morbidity, mortality, and healthcare resource use. In the United States, despite vaccination recommendations, the burden of VPDs remains high in adults aged 50 years and older. For instance, a projection study using a population-based modeling framework estimated that due to population growth and the shifting age distribution over the next 30 years, the annual societal economic burden for four major VPDs (influenza, pertussis, herpes zoster, and pneumococcal disease) is projected to increase from approximately $35 billion to $49 billion, resulting in cumulative costs of approximately $1.3 trillion, along with more than 1 million disease-related deaths (Talbird et al., 2021). For instance, older adults are more susceptible to pneumococcal infections. In Germany, data from 2005–2010 showed that seniors are prone to developing serious complications such as sepsis, especially during the winter months. A systematic review and meta-analysis indicated that despite pneumococcal vaccines being a proven preventive measure, the vaccination rate of older adults for this vaccine remains low in many primary healthcare settings (Ohta et al., 2025). For instance, a recent study showed that among senior patients diagnosed with invasive pneumococcal disease (IPD), only 26% had received any type of pneumococcal vaccine, and merely 16% had completed the vaccination within the past 5 years (Perniciaro and van der Linden, 2021), indicating a significant gap in prevention. Similarly, the prevalence of influenza-related hospitalizations and deaths is higher in older adult population. Although influenza vaccination is recommended, the vaccination rates are often suboptimal, and the efficacy of the vaccine can be reduced in this population due to immunosenescence (Korkmaz et al., 2019).

Vaccination rates among older adults vary significantly across the globe. In a study on COVID-19 vaccination programs for older adults, 192 countries reported their use of COVID-19 vaccines for this age group. Among countries with available data, the median proportion of individuals completing a primary series was 81% (IQR 58.3-92.0), for a first booster was 53% (14.1-81.7), for a second booster was 44.3% (13.5-69.7), and for a series vaccination was 23.6% (6.6-52.4), with large differences by region (Zheng et al., 2024). In Denmark, the uptake of influenza vaccines among older adults, both with and without dementia, showed an initial increase followed by a plateau from 2007/08 to 2018/19. However, the coverage was consistently below 60% during this period, not reaching the WHO target of 75%. Home-living older adults with dementia were 24% less likely to receive an influenza vaccine, highlighting an important target group for future vaccination programs (Appel et al., 2025). In the United States, efforts have been made to improve vaccination rates among rural older adults. An interprofessional academic detailing initiative, which included education and workflow assessment, increased pneumococcal vaccination rates among rural-dwelling older adults (McKeirnan et al., 2021). These global trends indicate the need for targeted strategies to improve vaccination coverage in the older adult population.

Therefore, this review aims to provide new insights into the challenges of vaccinating the older adult population and lay a theoretical foundation for formulating more effective immunization strategies targeting this vulnerable group.

## 2 Results

### 2.1 Research strategy

The following databases were investigated in an attempt to identify all the relevant studies published on PubMed, Web of Science, Google Scholar, the Cochrane Library and EMBASE. The literature search was conducted up to 14 July 2025. The topic search terms used were as follows: #1—“aging” OR “aging population” OR “older adults”; #2—“vaccine responsiveness” OR “vaccine efficacy” OR “vaccine reactivity” OR “responsiveness to vaccines”; #3—“inflammaging” OR “immunosenescence”; #4—#1 AND #2 AND #3; #5—#1 AND #2; #6—#2 AND #3.

### 2.2 Eligibility criteria

This review included published articles that reported vaccine responsiveness in older adults. The inclusion criteria were as follows: only studies published in English with clear indication of vaccination were included; only studies exploring the relationship between changes in immune function and vaccination in older adults were included. Meanwhile, case reports, case series, and review articles were also included, as were opinion articles and meta-analyses.

To explore the potential mechanisms underlying the impact of immunosenescence on vaccine responsiveness in aging population, this review investigated the mechanisms by which immunosenescence affects vaccines in older adults (aged ≥ 65 years), diagnostic techniques for assessing vaccine responsiveness, and therapeutic strategies to improve vaccine responsiveness in older adults.

### 2.3 Study selection

**FIGURE 1:**
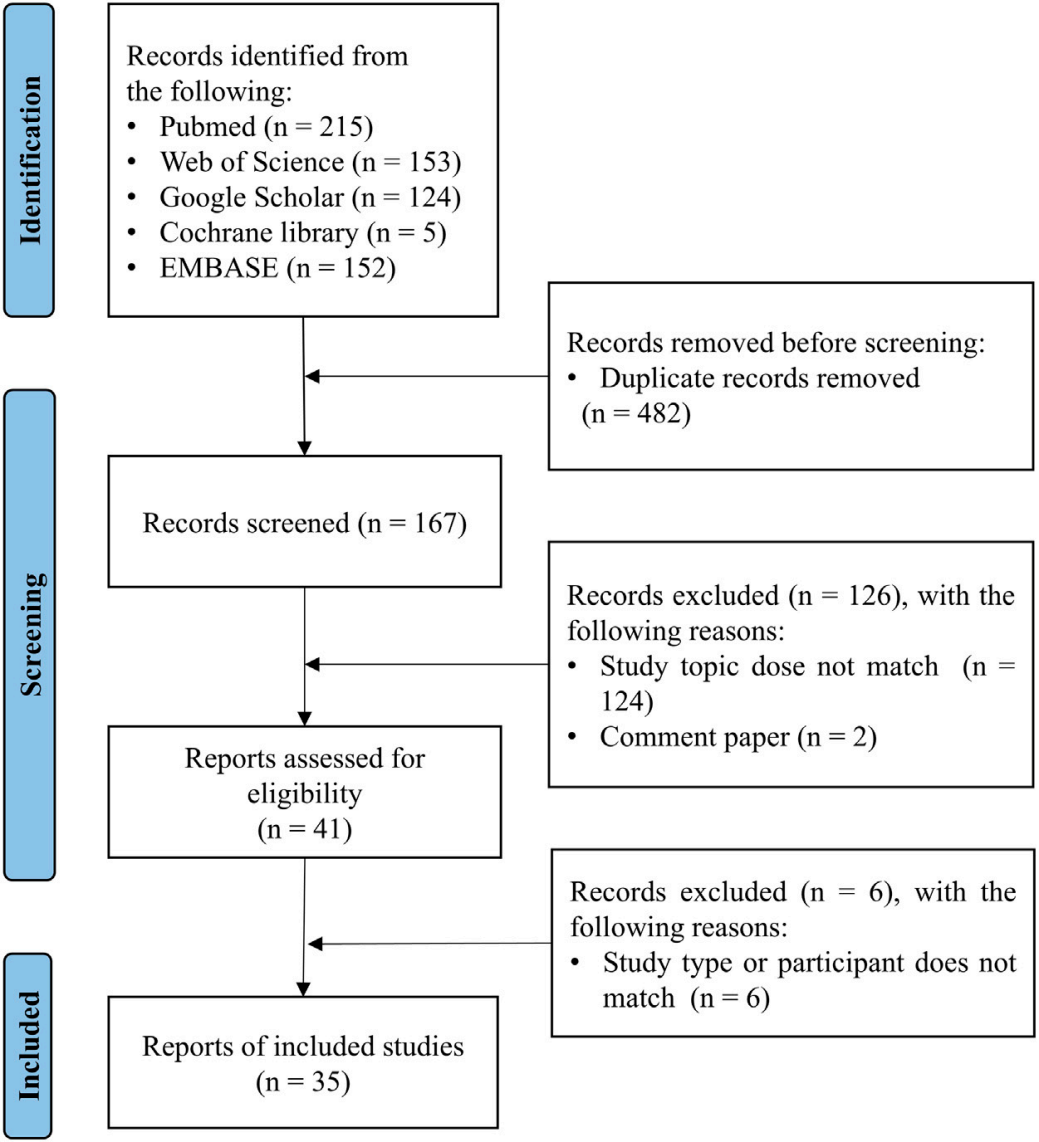
The process of study selection for vaccine responsiveness in aging population.

After manually removing duplicates, the titles and abstracts of the articles identified through the initial search were first screened. The full texts of the relevant articles were examined for the inclusion and exclusion criteria (Figure 1). After screening the abstracts, the full texts of the articles were assessed for eligibility and were selected or rejected for inclusion in the review. Any discordant results were discussed in a consensus meeting.

## 3 Immunosenescence and vaccine immune responsiveness

**FIGURE 2:**
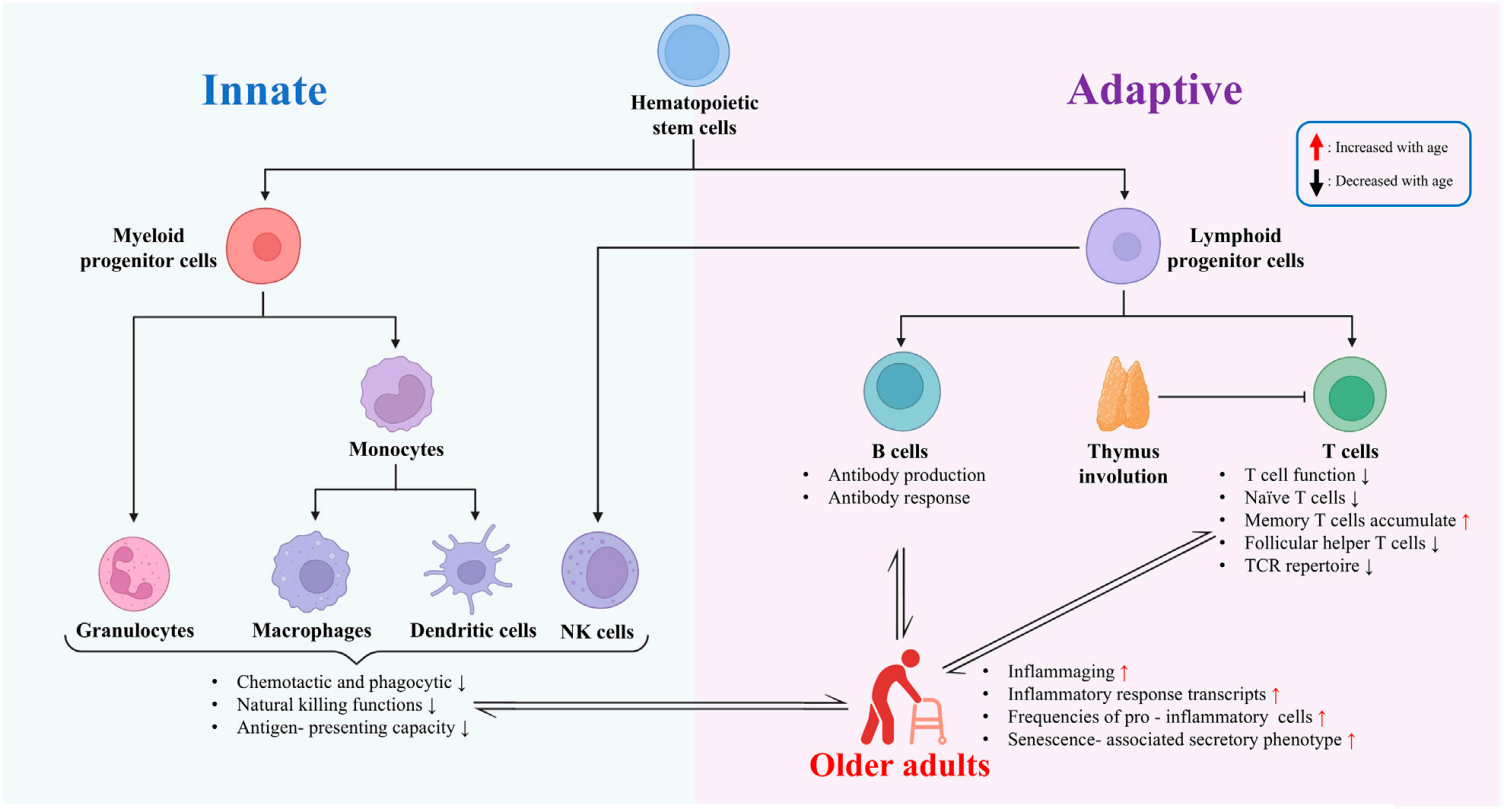
Immunosenescence and its impact on vaccine efficacy in older adults.

Immunosenescence refers to the phenomenon of age-related decline in immune system function, which significantly impacts vaccine-induced immune responses in older adults. This section first examines immunosenescence at the cellular, molecular, and signaling-pathway levels, and then discusses its impact on vaccine efficacy in older adults (Figure 2).

### 3.1 Age-related changes in immune system functionality

The aging process triggers multifaceted changes in the immune system, also known as immunosenescence. These changes involve declines in the function, proliferative capacity, quantity, and alterations in the proportion of cell subtypes of various cells in the innate immune system. Additionally, they affect the signal transduction pathways of immune cells, leading to impairments in immune cell clonal expansion, cytokine production, and differentiation functions. Ultimately, these changes collectively impact the body’s immune response to pathogens and vaccines. These age-related functional changes in the immune system serve as an important basis for understanding the decline in immune function in older adults.

#### 3.1.1 Cellular changes in the aging immune system

The aging process is accompanied by significant changes in both the innate and adaptive immune systems. In the innate immune system, there are alterations in chemotactic, phagocytic, and natural killing functions. Neutrophils, for instance, may exhibit reduced chemotaxis and phagocytosis, which are essential for the initial defense against pathogens (Lewis et al., 2022). Monocytes and macrophages also show functional changes, such as impaired antigen-presenting capacity and dysregulated inflammatory response. These changes can lead to a reduced ability to recognize and respond to pathogens effectively.

In the adaptive immune system, the most striking feature of immunosenescence is the decline in T cell function. There is a decrease in the proliferation of T cells, a reduction in the number of antigen-naïve T cells, and an increase in the number of antigen-experienced memory T cells. This shift is accompanied by a reduced overall TCR repertoire, which limits the ability of T cells to recognize a wide range of antigens (Song et al., 2025). At the single-cell level, transcriptome changes in T cells occur with age. For instance, genes related to T cell activation, proliferation, and cytokine production may be differentially expressed, leading to altered T cell functions. In CD8^+^ T cells, aged cells show decreased magnitude of responses, poor differentiation into effector cells, and a reduced arsenal of effector functions. This can be attributed to phenotypic and numeric changes to the naïve CD8^+^ T cell precursor pool, the impact of persistent viral infections and inflammation, and contributions of the aging environment in which these cells are activated (Nikolich-Žugich et al., 2012).

In the context of B cells, evidence suggests that the quantity of B cells tends to decrease with age in older adults; additionally, these cells may exhibit reduced antibody production and responsiveness, as well as alterations in cell memory (Frasca et al., 2020; Lewis et al., 2022). For instance, the ability to generate high-affinity antibodies may be impaired, which can affect the effectiveness of the humoral immune response to vaccines (Frasca et al., 2020; Lewis et al., 2022). Additionally, changes in the hematopoietic stem cell (HSC) compartment can also impact the immune system. Aging HSCs show a concomitant delay in differentiation and cell cycle, which can lead to a bias in the differentiation of immune cells and contribute to the deterioration of the immune system (Hérault et al., 2021).

#### 3.1.2 Molecular mechanisms of immunosenescence

Immunosenescence is associated with several molecular mechanisms that contribute to the decline of immune function and the reduced efficacy of vaccines in older adults. One of the key features is thymic involution, which leads to a decrease in the production of naïve T cells. As the thymus shrinks with age, the output of new T cells with a diverse T cell receptor (TCR) repertoire is reduced, limiting the ability of the immune system to respond to new antigens (Teissier et al., 2022; Liu et al., 2023). Additionally, chronic antigen stimulation, such as from persistent viral infections like cytomegalovirus (CMV), can mediate premature senescence of immune cells. As a long-term latent virus, CMV has a seroprevalence rate of up to 90% in the population aged ≥80 years (Staras et al., 2006). Its latent infection continuously activates the immune system, leading to elevated levels of pro-inflammatory cytokines such as interleukin-6 (IL-6) and tumor necrosis factor-α (TNF-α) in serum, thereby inducing a state of “inflammaging” (Franceschi et al., 2007; Wang et al., 2010). This chronic low-grade inflammation not only accelerates the functional decline of immune cells but also is directly associated with an increased incidence of frailty in older adults—studies have confirmed that the correlation between IL-6 levels and frailty phenotypes (e.g., slowed walking speed, decreased muscle strength) is significantly stronger in CMV antibody-positive older adults than in CMV antibody-negative ones (Schmaltz et al., 2005; Wang et al., 2010). Mechanistically, CMV can exacerbate the “blunting” of immune responses in older adults through two core pathways, ultimately resulting in insufficient immune responses: on one hand, CMV induces the accumulation of T cells in a terminally differentiated state, gradually depleting the immune system reserve; on the other hand, CMV disrupts the balance of cytokines, further aggravating immune dysfunction as well as down regulating vaccine responsiveness (Sawyer et al., 2012; van den Berg et al., 2019). This insufficient immune response is manifested in both humoral and cellular immunity, and is more prominent in older adults with comorbidities.

Senescent cells develop a pro-inflammatory senescence - associated secretory phenotype (SASP), which exacerbates inflammaging, a low - grade chronic inflammatory state associated with aging. Inflammaging, in turn, can interfere with the normal functioning of the immune system and reduce vaccine efficacy (Liu et al., 2023). Epigenetic alterations also play a role in immunosenescence. Changes in DNA methylation, histone modification, and non - coding RNA expression can affect gene activity and immune cell function. For instance, certain epigenetic changes may lead to the dysregulation of genes involved in T cell activation and differentiation, further contributing to the decline of immune function (Márquez et al., 2020; Liu et al., 2023).

#### 3.1.3 Impact of aging on immune cell signaling

Aging also affects the signaling pathways within immune cells, which can further disrupt immune function. In T cells, for instance, there are changes in TCR- and CD28^−^ dependent activation of downstream signaling effectors. Studies have shown that lymphocytes of senior subjects are already in an activated state that cannot be upregulated by external stimulation. The age-related signal transduction changes are more important than phenotype in CD4^+^ and CD8^+^ T cell subpopulations. For instance, the activity of kinases such as Lck, SHP-1, Akt, PI3K p85α, and mTOR, which are involved in T cell activation, is altered in older adults (Le Page et al., 2018).

These signaling changes can lead to a cascade of effects on T cell functions. Altered clonal expansion and decreased cytokine production, especially of IL-2, are common consequences. IL-2 is crucial for T cell proliferation and survival, and its reduced production can limit the ability of T cells to mount an effective immune response. In addition, changes in signaling pathways can also affect the differentiation of T cells into different subsets, such as effector and memory T cells, further impacting the overall immune response to infections and vaccines (Le Page et al., 2018). Understanding these age-related changes in immune cell signaling is essential for developing strategies to enhance immune function in older adults.

### 3.2 Immunosenescence and inflammaging: their impact on vaccine responsiveness

Immunosenescence, the age-related decline of the immune system, significantly impacts vaccine-induced immune responses in older adults. As individuals age, their physiological functions deteriorate, including a compromised immune response, which leads to an increased susceptibility to diseases and a reduced effectiveness of vaccines (Chen et al., 2024). For instance, studies have shown that the efficacy of standard influenza vaccines is only 30%–50% in the older population (Wagner and Weinberger, 2020). This is due to a variety of factors, such as various changes of T cells and B cells associated with immunosenescence, which is mentioned above.

The impact of immunosenescence on vaccination is not limited to antibody responses. T cell mediated responses are also reshaped during aging. The decline in the naïve T - cell population is associated with impaired immunity and a reduced response to new or mutated pathogens (Lewis et al., 2022),with the accumulation of memory T cells, significantly impact vaccine responsiveness in aging population (Crooke et al., 2019). Also, in older adults, T cells tend to favor the generation of short - lived effector T cells rather than memory or follicular helper T (TFH) cells. This imbalance can result in vaccine - induced antibodies with lower protective capacity (Gustafson et al., 2020).

Additionally, the innate immune system also undergoes changes with age. For instance, the function of dendritic cells, which are important for antigen presentation, is impaired. This can affect the priming of adaptive immune responses, further compromising vaccine efficacy (Grubeck-Loebenstein et al., 2009). Moreover, a low - level chronic inflammatory state known as inflammaging has been confirmed to be interrelated with immunosenescence, and the two jointly participate in the process of inhibiting vaccine immune responses.

From the perspective of mechanistic association, immunosenescence serves as a crucial initiator of inflammaging, and inflammaging in turn exacerbates immunosenescence through the establishment of a chronic inflammatory microenvironment, thereby forming a vicious cycle. A study confirmed by specific knockout of DNA repair genes in mouse hematopoietic cells showed that senescent immune cells (e.g., T cells, B cells, NK cells) can release pro-inflammatory signals by secreting senescence-associated secretory phenotype (SASP) substances (such as IL-6 and CCL2). This not only directly induces DNA damage and upregulation of senescence markers (p16, p21) in non-lymphoid organs like the liver and kidneys, but also amplifies the systemic inflammatory state through a “non-cell-autonomous” mechanism (Yousefzadeh et al., 2021). Another study indicated that IL-11, whose expression increases with age, can promote the processes of immunity-related senescence and inflammaging by activating the ERK-p90RSK/mTORC1 pathway. The specific manifestations include upregulated expression of the senescence marker p16 in immunity-related tissues (e.g., the spleen) and a significant increase in the serum level of the inflammatory factor IL-6. However, intervention measures such as knockout of IL-11-related genes or injection of anti-IL-11 antibodies can simultaneously alleviate the above two senescent phenotypes, which not only significantly reduce the level of inflammatory factors in aged mice but also improve the function of their immune cells (e.g., restoring the proliferation potential of T cells) (Widjaja et al., 2024). In addition, a study found that the activation of the NLRP3 inflammasome in dendritic cells (DCs) of aged mice can promote the release of IL-1β. This process not only exacerbates the body’s inflammatory state (e.g., increased serum levels of inflammatory factors) but also impairs the migration ability of DCs to lymph nodes due to insufficient expression of CCR7 on the DC surface, thereby affecting the immune function of aged mice (Zhivaki et al., 2024). In the intricate and extensively interconnected regulatory network of immunosenescence and inflammaging, a number of clinical trials in recent years have indicated that the mTOR signaling pathway may serve as a core node mediating the interaction between these two processes (Mannick et al., 2018; Mannick et al., 2021). Specifically, the mTOR-S6K signaling pathway can regulate inflammaging by modulating immune receptors through the endolysosomal system; at the same time, this pathway can also ameliorate the state of immunosenescence, which is specifically manifested by the enhancement of the body’s pathogen clearance ability (Zhang et al., 2024). The aforementioned effects enable the mTOR-S6K signaling pathway to establish a bidirectional association between immunosenescence and inflammaging—two key hallmarks of aging. Moreover, the regulation of both processes is dependent on the involvement of the mTOR inhibitor (Mannick et al., 2018; Mannick et al., 2021; Zhang et al., 2024), and the specific mechanisms thereof will be elaborated in the following text.

In summary, on the one hand, immunosenescence can drive the occurrence of inflammaging in older adults through such pathways as myeloid-biased differentiation of hematopoietic stem cells, depletion of tissue-resident macrophages, and impaired function of immune effector cells. Specifically, it constructs local and systemic pro-inflammatory microenvironments by releasing pro-inflammatory factors including IL-1 and IL-11. On the other hand, this inflammatory microenvironment further amplifies the immunosuppressive phenotypes associated with immunosenescence, such as inhibiting NK cell activity and promoting the accumulation of myeloid-derived suppressor cells, thus forming a positive feedback loop of “immunosenescence-inflammaging”. This loop then directly or indirectly affects the vaccine response in older adults (Yousefzadeh et al., 2021; Park et al., 2024; Widjaja et al., 2024; Zhivaki et al., 2024; Huang et al., 2025).

With the increase of age, the number of senescent cells increases in older adults (Marrella et al., 2022). Cellular senescence acts as a key link connecting immunosenescence and inflammaging: on the one hand, cellular senescence can directly promote inflammaging by enhancing inflammatory responses (Sanada et al., 2018); on the other hand, the impaired clearance of senescent cells caused by immunosenescence leads to the continuous accumulation of senescent cells, which further exacerbates immunosenescence (Huang et al., 2025). Meanwhile, as mentioned earlier, a positive feedback loop can be formed between immunosenescence and inflammaging. Specifically, immunosenescence is characterized by reduced phagocytic activity of dendritic cells, decreased production of naive T cells with accumulation of antigen-experienced T cells, reduced number of naive T cells with impaired TCR signaling, altered differentiation tendency of T cells, and impaired B cell function. It affects the immune response of older adults at multiple levels, including innate immunity, cellular immunity, and humoral immunity, while promoting inflammaging. In contrast, inflammaging can not only directly affect the vaccine response of older adults but also indirectly impact their immune response by promoting immunosenescence (Park et al., 2024; Falahi et al., 2025; Huang et al., 2025). As indicated by the aforementioned clinical trials on mTOR inhibitors—agents capable of simultaneously suppressing both immunosenescence and inflammaging—the older adult population receiving injections of this drug exhibited a significant improvement in their immune response to the influenza vaccine (Mannick et al., 2018).

## 4 Strategies to predict and enhance vaccine responsiveness in older adults

In the preceding sections, we discussed how immunosenescence influences the immune response to vaccines in older adults. In the present section, we will address the prediction and enhancement of vaccine immune responses in this older adults population. The discussions on “age-related changes at the cellular level, abnormalities in molecular mechanisms, and alterations in signaling pathways” in Section 3 of this article can be further investigated with reference to the “vaccine responsiveness prediction techniques” discussed in Section 4, thereby fully exploring their potential value as biomarkers for predicting immune responsiveness in older adults. This linkage logic of “mechanism-technology” is precisely the core connection between Section 3 and Section 4. We also enumerate the strategies for enhancing the immune response in older adults, and match the corresponding intervention strategies to the specific immunosenescence-related changes mentioned in Section 3. Based on these fundamental linkages, this article aims to provide readers with more targeted insights for future research topic selection in the field of geriatric vaccine immunology.

### 4.1 Predictive techniques for assessing vaccine responsiveness

This section focuses on biomarkers for predicting vaccine responses in older adults and advanced immunological assays for evaluating vaccine efficacy. With respect to predictive biomarkers, specific T cell subsets, B cell-related factors, and inflammatory markers, can predict vaccine responsiveness in older adults, providing information for personalized vaccination strategies (Fourati et al., 2016; Lingblom et al., 2018; Cevirgel et al., 2025). Extensive research has been conducted in this field, covering multiple dimensions such as various vaccine types (e.g., pneumococcal vaccines, influenza vaccines) and different populations (e.g., healthy individuals, patients with lupus) (Zapata and HIPC-I Consortium, 2017; Kotliarov et al., 2020; Ravichandran et al., 2024). In terms of advanced immunological assays, the *in vitro* overlay assay, mycobacterial growth inhibition assay (MGIA), and serological assays are likely to become research priorities in the future due to their high accuracy (Tanner et al., 2016; Tsang et al., 2020; Pasupuleti et al., 2024).

#### 4.1.1 Biomarkers for predicting vaccine responsiveness in older adults

Identifying biomarkers associated with immunosenescence and vaccine responsiveness is crucial for predicting vaccine efficacy and developing personalized vaccination strategies in older adults. Studies investigating T cell phenotypes have revealed that in low vaccine responders from the VITAL cohort, the percentages of CD31^+^naïve CD4^+^, CD31^+^naïve CD8^+^, and CD38^+^ naïve CD8^+^ T cells are significantly lower; these naïve T cell subsets show a stronger correlation with immune entropy (a measure of cumulative immune system perturbations) than with age itself, suggesting their association with weak vaccine responsiveness and immunosenescence (Cevirgel et al., 2025). Additionally, a study using mass cytometry (CyTOF) to characterize circulating immune cell populations in older adults before and after administration of an investigational adjuvanted protein vaccine against respiratory syncytial virus (RSV) found baseline differences between responders and non-responders, with non-responders exhibiting higher levels of activated (HLA-DR^+^) CD4^+^ and CD8^+^ T cells, indicating these cell populations may relate to differential vaccine responsiveness (Lingblom et al., 2018; Nehar-Belaid et al., 2023).

Beyond T cell biomarkers, investigations into B cell factors have shown that in studies on hepatitis B and influenza vaccination in older adults, heightened expression of genes augmenting B cell responses and higher memory B cell frequencies correlate with stronger vaccine responses, while higher levels of inflammatory response transcripts and increased frequencies of pro-inflammatory innate cells correlate with weaker responses (Fourati et al., 2016). Collectively, these findings indicate that a combination of T cell and B cell related biomarkers, along with inflammation markers, may be useful for predicting vaccine responsiveness in older adults, potentially informing targeted strategies such as adjusting vaccine formulations or schedules based on individual immune profiles.

#### 4.1.2 Advanced immunological assays for vaccine efficacy

Advanced immunological assays play a vital role in accurately assessing vaccine efficacy. One such assay is the *in vitro* overlay assay, which can be used to evaluate the immunogenic properties of early-stage vaccine formulations. This assay allows for the quantification of T cell proliferation upon successful antigen presentation by vaccine-stimulated dendritic cells. For instance, when testing nanoparticulate vaccine formulations targeting various pathogens, the assay revealed robust T cell proliferation in the vaccine treatment groups, with variations between bacterial and viral vaccine candidates. A dose-dependent study also indicated that immune stimulation varied with antigen dose, highlighting the assay’s potential to differentiate and quantify effective antigen presentation (Pasupuleti et al., 2024). Another important assay is the MGIA, which is used to assess protective immunity and evaluate tuberculosis vaccine efficacy. Unlike measurements of single immunological parameters, MGIAs represent an unbiased functional approach that takes into account a range of immune mechanisms and their complex interactions. This controlled model not only enables rigorous assessment of vaccine efficacy and dissection of protective immune mediators against *Mycobacterium tuberculosis* (Tanner et al., 2016), but also serves as a valuable reference for analogous studies of other bacterial and viral pathogens. Additionally, serological assays are commonly used to monitor antibody responses to vaccines. For instance, in the evaluation of single-dose HPV vaccines, seven different serological assays were compared. The results supported the utility of existing serological assays to monitor antibody responses following single-dose HPV vaccination, as they showed good reproducibility and validity in measuring HPV - 16/18 immunological responses (Tsang et al., 2020).

### 4.2 Strategies to enhance vaccine responsiveness

Following the exploration of how to predict vaccine responsiveness in older adults, enhancing such responsiveness is of greater practical significance and importance. Relevant content such as increasing the antigen dose in vaccines and altering vaccine injection routes has been reviewed in other literatures (Pereira et al., 2020; Soegiarto and Purnomosari, 2023). Therefore, in this section, we summarize the current mainstream theories for improving vaccine responses in older adults, which mainly include the use of adjuvants, immunomodulation, anti-inflammation, and targeting T-cell aging, and discuss their current applications in practice (Table 1). We also match the corresponding intervention strategies to the specific immunosenescence-related changes mentioned in Section 3 (Table 2).

**TABLE 1:** Strategies to enhance vaccine responsiveness in older adults.

Type	Example	Function	Reference
Adjuvant based on oil - in - water emulsions	MF59™ and AS03	Enhancing the immunogenicity of vaccine antigens leads to a stronger immune response	Weinberger (2018)
Adjuvant based on virosomes	Virosomes	Enhancing the immunogenicity of vaccine antigens leads to a stronger immune response	Weinberger (2018)
Adjuvant based on liposomes	AS01	Boost APC activation and initiate T and B cell response	Weinberger (2018)
Adjuvant based on Cytokines	IL-2, IL-7, IL-15, IL-12, IL-21 and GM-CSF	Enhance immune response and increase vaccine efficiency	Cao et al. (2024)
Immunomodulatory	Inhibit mTOR protein kinase	Reduce infection rates, upregulate antiviral gene expression, and improve vaccine response	Mannick et al. (2018)
Anti - inflammatory	Anti – inflammatory drugs	Reverse the suppression of vaccine response by inflammaging	Pereira et al. (2020)
Target T - cell aging	Rejuvenate T - cell populations	Potentially enhance immune resilience improve vaccine efficiency	Chen et al. (2025)
	Modify immunological landscape	Potentially enhance immune resilience improve vaccine efficiency	Chen et al. (2025)

**TABLE 2:** Matching specific immunosenescence changes with intervention strategies.

Immunosenescence changes	Intervention strategies	Mechanism	Reference
Signaling pathway defects Increased activation threshold Impaired proliferation Abnormal cytokine production Abnormal subset differentiationof T cell	1. IPMPs 2. mTORC1 inhibitor combination regimen (BEZ235 + RAD001) 3. AS01 4. Strategies targeting T cell aging	1. IPMPs can elicit more Th1-polarized immune responses 2. Inhibitors inhibit excessive activation of mTORC1, which can restore T cell differentiation and signal balance 3. Promote the initiation of T cell responses 4. Therapies to rejuvenate T cell populations and adjust the immune landscape are currently under exploration	Zhang et al. (2015), Mannick et al. (2018), Weinberger (2018), Chen et al. (2025)
Abnormal antibody production and memory function of B cells	AS01	Enhance the activation and antigen presentation of DCs, which can promote the initiation of B cell responses	Weinberger (2018)
Inflammaging (chronic low - grade inflammation)	Anti-inflammatory drugs	Reduce the level of basal inflammation, and relieve it’s inhibition on vaccine-induced immune responses	Pereira et al. (2020)
Decreased antigen - presenting capacity of DCs	1. Oil-in-water emulsion adjuvants (MF59/AS03) 2. IPMPs 3. AS01	1. Promote the recruitment and maturation of DCs, and improve antigen presentation efficiency 2. Significantly enhance the efficacy on BMDCs and pMΦs 3. Enhance the activation of antigen-presenting cells such as dendritic cells	Zhang et al. (2015), Turley and Lavelle (2022) Weinberger (2018)

#### 4.2.1 Adjuvant - based approaches

Adjuvants play a crucial role in enhancing the efficacy of vaccines in older adults. They work by activating the innate immune system, thereby enhancing the magnitude, functionality, breadth, and durability of immune responses (Turley and Lavelle, 2022). For instance, the oil - in - water emulsions MF59TM and AS03, as well as a virosomal vaccine, have been licensed in seasonal or pandemic influenza vaccines and have been used successfully in older adults. These adjuvants can increase the immunogenicity of the vaccine antigens, leading to a stronger immune response (Weinberger, 2018).

Another example is the liposome - based adjuvant AS01, which comprises two immunostimulants. It has been approved in a recombinant protein vaccine for older adults and showed very high efficacy against herpes zoster in clinical trials. AS01 can enhance the activation of antigen presenting cells, such as dendritic cells, and promote the priming of T cell and B cell responses (Weinberger, 2018). In addition to these licensed adjuvants, several others are in clinical and pre - clinical development. For instance, some studies are exploring the use of cytokine - based adjuvants. Cytokines like Type I interferons, Type II IFN, interleukins (e.g., IL-2, IL-7, IL-15, IL-12, and IL-21), and granulocyte-macrophage colony-stimulating factor (GM-CSF) have shown promise in boosting immune responses and enhancing vaccine efficacy in animal models of tuberculosis and other diseases (Cao et al., 2024).

Adjuvant development is a key strategy to enhance vaccine efficacy, especially in older adults. New adjuvants are being designed to overcome the limitations of traditional adjuvants and better target the age - related changes in the immune system. For instance, immunopotentiator - loaded polymeric microparticles have shown promise as robust adjuvants. In a study, imiquimod (TLR-7 ligand)-loaded PLGA microparticles (IPMPs) were prepared. Incorporating imiquimod into microparticles significantly improved the efficacy of PLGA microparticles in activating bone marrow - derived dendritic cells (BMDCs) and peritoneal macrophages (pMΦs), and promoted antigen uptake by pMΦs. IPMPs showed stronger adjuvanticity to augment OVA-specific immune responses compared to plain PLGA microparticles, and elicited more Th1-polarized immune responses (Zhang et al., 2015).

Another approach is the development of QS-21 based vaccine adjuvants. QS-21, a saponin based vaccine adjuvant, has potent immunostimulatory properties but is limited by scarcity, complex synthesis, and toxicity. Researchers have developed QS-21 analogues, such as VA05 and VA06, with simplified structures, enhanced immunogenicity, and reduced toxicity. The antibody titers generated by VA05 and VA06 in conjunction with the antigen were comparable to those induced by QS-21, making them promising candidates for next-generation adjuvant development (Luo et al., 2025). Additionally, cytokine - based adjuvants are being explored. As mentioned above, cytokines, interleukins and GM - CSF have shown effectiveness in boosting immune responses and enhancing vaccine efficacy in tuberculosis models (Cao et al., 2024).

#### 4.2.2 Immunomodulatory therapies

Immunomodulatory therapies offer another approach to boost vaccine responses in older adults. These therapies can target various aspects of the immune system to enhance its function. For instance, mTOR inhibitors, with their unique property of simultaneously improving immunosenescence, inflammaging, and vaccine responsiveness in older adults without interfering with adjuvant-induced local inflammation, are expected to become a key focus of future research. In a phase 2a randomized, placebo-controlled clinical trial, a low dose combination of a catalytic (BEZ235) plus an allosteric (RAD001) mTOR inhibitor that selectively inhibits target of rapamycin complex 1 (TORC1) downstream of mTOR was associated with a significant (P = 0.001) decrease in the rate of infections reported by senior subjects for a year after study drug initiation. It also led to an upregulation of antiviral gene expression and an improvement in the response to influenza vaccination in this treatment group (Mannick et al., 2018). In the phase 2b and phase 3 randomized trials of the oral mTOR inhibitor RTB101 (formerly known as BEZ235) conducted in adults aged 65 years and older, Mannick et al. found that RTB101 enhances antiviral immunity through the interferon (IFN)-mediated antiviral pathway, thereby improving immunosenescence and alleviating infection-driven inflammaging (Mannick et al., 2021). Zhang et al. extended the above findings to the mechanistic level, revealing how S6 kinase (S6K), a downstream effector of mTOR, regulates inflammaging and immunosenescence through the endolysosomal system, as well as the impact of this process on lifespan extension (Zhang et al., 2024). The study showed that S6K inhibition can upregulate the SNARE protein Syntaxin 13 (Syx13); Syx13 maintains endolysosomal integrity by preventing the accumulation of multilamellar lysosomes, thereby promoting the degradation of the regulatory isoform of the IMD receptor PGRP-LC (a driver of age-related sterile inflammation) (Zhang et al., 2024). Meanwhile, fat body-specific S6K activation blocks the enhancing effect of rapamycin (an mTOR inhibitor) on the bacterial clearance ability of aged *Drosophila* (Zhang et al., 2024).

Another immunomodulatory approach is the use of anti-inflammatory drugs. As mentioned earlier, inflammaging can inhibit immune responses to vaccination. Pharmacological strategies aiming at blocking baseline inflammation, such as the use of anti-inflammatory drugs, could potentially boost vaccine responses, but more research is needed to understand the role of inflammation in vaccine responses and to reconcile the seemingly paradoxical observations of using adjuvants to promote local inflammation while trying to counteract inflammaging—given that non-selective, high-dose, and long-term use of systemic anti-inflammatory therapy may interfere with the local pro-inflammatory effects of adjuvants and even exacerbate immunosuppression (Pereira et al., 2020).

The local pro-inflammatory effect of adjuvants depends on the activation of innate immune pathways (e.g., TLR-NF-κB pathway, MAPK pathway): specifically, MF59 promotes antigen presentation by recruiting DCs and activating local cytokines (e.g., IL-1β, TNF-α); TLR agonists enhance T cell responses by activating TLR signaling (Casella and Mitchell, 2008; Coffman et al., 2010). However, non-selective anti-inflammatory drugs (e.g., NSAIDs, high-dose glucocorticoids) extensively inhibit such pathways shared by local and systemic inflammation (Reed et al., 2013; Henson et al., 2015; Vukmanovic-Stejic et al., 2018). This “non-selective pathway inhibition” directly prevents adjuvants from effectively initiating local immune activation, thereby interfering with their pro-inflammatory effects (Pereira et al., 2020). In addition, immune cells in older adults already have functional defects due to aging (e.g., loss of T cell proliferation capacity, insufficient B cell antibody production), and high-dose, long-term systemic anti-inflammatory therapy further exacerbates immunosuppression (Pereira et al., 2020).

In contrast, selective, short-term, low-dose anti-inflammatory therapy can avoid interfering with adjuvant effects: specifically, short-term use of the p38 MAPK inhibitor Losmapimod (which only targets the p38 pathway mediating systemic inflammaging) improves the response of older adults to cutaneous VZV-specific antigens without affecting the adjuvant-induced local T cell activation (Vukmanovic-Stejic et al., 2018); low-dose use of the mTOR inhibitor increases the antibody titer induced by influenza vaccines in individuals aged ≥65 years by more than 20%, while preserving the local pro-inflammatory effect of the MF59 adjuvant (Mannick et al., 2014). Therefore, clarifying the differential regulatory mechanisms between adjuvant-induced local inflammation and systemic inflammation associated with inflammaging is of great significance for optimizing vaccine response strategies in older adults.

At present, mTOR inhibitors and p38 kinase inhibitors have obtained support from some clinical studies. Both exhibit promising potential in improving immunosenescence, inflammaging, and vaccine responsiveness in senior patients, and are expected to become clinical medications in relevant fields. Among them, mTOR inhibitors show good efficacy in improving multiple phenotypes of aging, and may become a key research direction in this field in the future. Additionally, strategies that target T cell aging, such as therapies to rejuvenate T cell populations and modify the immunological landscape, are being explored. These could potentially enhance immune resilience in older adults and improve the efficacy of vaccines (Chen et al., 2025).

#### 4.2.3 Other therapies

In addition to adjuvants and immunomodulatory therapies, the following therapies also hold clinical application potential for improving immunosenescence, inflammaging, and vaccine response in older adults. Strategies that target T cell aging, such as therapies to rejuvenate T cell populations (including metabolic intervention, adoptive cell therapy, targeted therapy) and modify the immunological landscape, are being explored (Chen et al., 2025). These could potentially enhance immune resilience in older adults and improve the efficacy of vaccines (Chen et al., 2025). Our previous study revealed that insufficient epitope-specific T cell clones lead to impaired immune responses to the SARS-CoV-2 vaccine in older adults, which also supports this viewpoint (Xiao et al., 2023).

Regarding the complex relationships among cellular senescence, immunosenescence, and inflammaging mentioned earlier, senolytic therapy can selectively eliminate senescent cells, reduce the exacerbation of immunosenescence and inflammaging caused by the SASP secreted by senescent cells, and decrease the inflammatory burden by clearing senescent cells, thereby enhancing the immunogenicity of vaccines in older adults (Falahi et al., 2025). The main approaches of senolytic therapy include: using senescent cell-clearing agents such as CAR-T cells, dasatinib combined with quercetin, and fisetin (Choi et al., 2023; Amor et al., 2024; Chen et al., 2025); SGLT2 inhibitors (e.g., canagliflozin) exerting a senescent cell-clearing effect by activating AMPK activity (Katsuumi et al., 2024); PD-1 antibodies reducing the accumulation of PD-L1^+^ senescent cells while enhancing the activity of CD8^+^ CTLs, and they can also be used in combination with senescent cell-clearing therapy to improve efficacy (Wang et al., 2022).

In addition, age-related gut microbiota dysbiosis in older adults—such as a reduction in beneficial bacteria and an increase in pro-inflammatory bacteria—can disrupt the intestinal mucosal barrier, leading to the translocation of gut bacteria and their metabolites into the bloodstream and inducing chronic inflammation. However, gut microbiota modulation, such as probiotic supplementation, can restore gut microbiota balance, reduce the release of inflammatory factors, and improve immune function. Studies have indicated that probiotics have shown positive effects in parenteral influenza vaccination and oral vaccination in older adults, and can assist in enhancing vaccine-induced immune responses (Zimmermann and Curtis, 2018; Falahi and Kenarkoohi, 2022).

Non-pharmacological approaches can also delay immunosenescence by regulating the body’s metabolic and immune status, thereby improving vaccine responsiveness in older adults. For example, caloric restriction can downregulate PLA2G7 to reduce inflammation and improve thymopoiesis; regular exercise can decrease the levels of T-cell senescence markers; supplementation with NAD^+^ precursors can maintain cellular metabolic vitality; and fasting-mimicking diets can induce ketone body production and enhance CD8^+^ T-cell function (Chen et al., 2025).

### 4.3 Novel vaccine formulations for the aging population

Novel vaccine formulations are being developed to better address the immunosenescence-related challenges in older adults. One example is the development of a primary meningococcal vaccine for middle aged adults. A tetravalent meningococcal vaccine conjugated to tetanus toxoid (MenACWY - TT) was administered to middle aged adults (50–65 years of age). The vaccine clearly induced naïve responses to meningococci W and Y, and booster responses to meningococci C. After 28 days, a high percentage of participants possessed a protective serum bactericidal assay (SBA) titer for MenC, MenW, and MenY, which was maintained in a significant proportion of participants 1 year post - vaccination. The SBA titers correlated well with the meningococcal - specific IgM responses, especially for MenW and MenY, and these IgM responses were negatively correlated with age. This suggests that primary immunization with this vaccine in middle - aged adults can induce long - lasting protective antibody titers and that vaccination before reaching old age may be beneficial (van der Heiden et al., 2017).

Another approach is the use of adjuvanted influenza vaccines. MF59-adjuvanted vaccines, designed to improve the immune response in older adults, have been used in clinical practice since 1997 in their trivalent formulation and since 2020 in their tetravalent formulation. Data from various studies show that these vaccines are safe for all age groups, with a reactogenicity profile similar to that of the conventional vaccine. They are especially effective in boosting the immune response in the population aged 65 or over by increasing antibody titers after vaccination and significantly reducing the risk of hospital admission (Arrazola Martínez et al., 2023). Additionally, a recombinant vaccine containing the viral glycoprotein gE and the novel adjuvant AS01B has shown high efficacy against herpes zoster, even in the oldest age groups, after administration of two doses (Wagner and Weinberger, 2020).

## 5 Discussion

Conducting vaccine trials in older adults raises several ethical considerations. One major concern is the balance between the potential benefits of the vaccine and the risks to the participants. For instance, in the case of influenza vaccination, while there is a need to determine the true efficacy of the vaccine in reducing mortality in older adults, conducting a placebo - controlled trial may be ethically questionable. Withholding vaccination from the control group could substantially increase their risk for influenza and its complications, as vaccination is considered competent care. Given the high burden of disease and already proven benefits of vaccination, such a trial may not meet the Declaration of Helsinki, which states that the importance of a trial must outweigh the risk patients are exposed to (Verhees et al., 2018).

Another ethical consideration is the issue of informed consent. Senior participants may be more vulnerable due to cognitive decline or comorbidities, making it crucial to ensure that they fully understand the nature, risks, and benefits of the vaccine trial. Additionally, there is a need to ensure that the trial design is fair and does not disproportionately affect certain subgroups of older adults. For instance, in resource - constrained settings, there is a risk that trials may exploit vulnerable populations. Therefore, careful consideration must be given to the selection of participants, the provision of adequate healthcare, and the communication of trial results to ensure that the rights and wellbeing of the senior participants are protected (Moodley et al., 2013).

Future research in immunology and aging should focus on several key areas. One important direction is to further understand the mechanisms of immunosenescence at the molecular and cellular levels. This includes investigating the role of epigenetic changes, mitochondrial dysfunction, and the impact of persistent viral infections on immune cell function. By elucidating these mechanisms, it may be possible to develop more targeted interventions to reverse or mitigate immunosenescence and improve vaccine responsiveness (Wang et al., 2025). Another area of research is the development of personalized vaccination strategies. This could involve identifying biomarkers that can accurately predict an individual’s response to a vaccine, allowing for the customization of vaccine formulations, dosages, and vaccination schedules. For instance, by understanding an individual’s immune profile, it may be possible to select the most appropriate vaccine and adjuvant combination to maximize vaccine efficacy (Montin et al., 2024). Lastly, the dimension of sex medicine should not be overlooked either. Studies have indicated that even in older populations, where immunosenescence (a state of age-related immune decline) universally impairs vaccine responses, sex-based differences remain clinically relevant. After receiving influenza and COVID-19 vaccines, older women (≥65 years) still exhibit higher antibody titers than age-matched men, although the magnitude of this difference is slightly reduced due to age-associated declines in estradiol levels (Potluri et al., 2019; Calabrò et al., 2023). More importantly, older women also face a higher risk of vaccine adverse events: in a study on COVID-19 vaccination among residents of long-term care facilities, the proportion of women reporting local adverse events was higher than that of men, even after adjusting for comorbidities (Calabrò et al., 2023; Yin et al., 2024). These persistent differences highlight the necessity of developing sex medicine-integrated vaccination strategies. For instance, vaccine doses can be adjusted (e.g., administering half-dose vaccines to women under resource-constrained conditions) to balance vaccine efficacy and the risk of adverse events; alternatively, estrogen-centered interventions (such as hormone replacement therapy) can be prioritized for postmenopausal women to maintain their vaccine responsiveness (Calabrò et al., 2023; Scully et al., 2025). Additionally, adopting sex-sensitive communication approaches—acknowledging the higher risk of adverse events faced by women and providing targeted guidance (e.g., recommendations for vaccination scheduling, pain management methods)—helps alleviate vaccine hesitancy, as concerns about adverse events are a key factor leading to delayed or missed vaccinations among women (Calabrò et al., 2023; Scully et al., 2025). Addressing the manifestation of sex differences in vaccine research is not only a requirement for scientific rigor but also a critical step toward achieving equity in vaccination strategies, thereby ensuring that vaccines exert optimal effects for all populations. Finally, considering the complex associations among cellular senescence, immunosenescence, and inflammaging—along with the characteristic that these associations can directly or indirectly affect vaccine responses in older adults—anti-aging drugs with the function of selectively eliminating senescent cells could be explored as novel pharmaceutical intervention approaches in future vaccinology research to enhance the immunogenicity of vaccines in older adults (Soegiarto and Purnomosari, 2023).

Furthermore, research should focus on improving the delivery of vaccines to older adults. This could include developing novel vaccine delivery systems, such as nanoparticles or mucosal delivery platforms, to enhance vaccine uptake and immune response. Finally, there is a need for more research on the long - term effects of vaccination in older adults, including the durability of immune responses and the potential for vaccine - associated adverse events over time.
